# Characterization and Treatment Patterns of Peri/Menopausal and Postmenopausal Women with and Without Vasomotor Symptoms in a Retrospective Database Study

**DOI:** 10.1177/26884844251366113

**Published:** 2025-08-08

**Authors:** Lena Charafi, Kristina Bolling, Bridgette Kanz Schroader, Lisa Halvorson

**Affiliations:** ^1^ Bayer HealthCare, Whippany, NJ, USA.; ^2^ Cencora, Conshohocken, PA, USA.

**Keywords:** vasomotor symptoms, treatment patterns, asymptomatic menopause, symptomatic menopause, health disparities

## Abstract

**Introduction:**

Vasomotor symptoms (VMS) are the most commonly reported menopausal symptoms and vary across race and ethnicity, with higher prevalence and duration among Black and Hispanic women. This study describes demographics/clinical characteristics and treatment patterns of peri/menopausal and postmenopausal women with symptomatic menopause/VMS and asymptomatic menopause in a commercial claims population.

**Methods:**

Data from Optum’s deidentified Clinformatics Data Mart Database were obtained for peri/menopausal (aged 40–54 years) and postmenopausal (aged 55–64 years) women. VMS is not available directly in claims data and was proxied using symptomatic menopausal ICD-9/10 codes. In Phase 1, prevalence of symptomatic and asymptomatic peri/menopause (defined by ICD-9/10 codes) and baseline demographics/clinical characteristics were obtained. Phase 2 included baseline demographics/clinical characteristics, symptomatic menopause/VMS incidence rates, and treatment patterns.

**Results:**

Phase 1 included 1,987,355 ICD-9/10 codes for symptomatic or asymptomatic menopause. Peri/menopausal women had lower symptomatic menopause/VMS prevalence compared to postmenopausal women (6.5% vs. 4.9%). Symptomatic menopause/VMS prevalence was 5.7% and highest in White (6.3%) and lowest in Asian (3.4%) women.

Phase 2 included 203,546 (53.3%) peri/menopausal and 178,658 (46.7%) postmenopausal women. Symptomatic menopause/VMS incidence was 1.2%; only 52.9% were treated. Lower incidence and treatment rates were seen among Asian (0.46%; 33.2%), Hispanic (0.43%; 46.7%), and Black (0.41%; 47.1%) women compared to White (0.59%; 55.5%) women.

**Conclusions:**

This study adds to the literature by characterizing women with symptomatic menopause/VMS across the menopausal spectrum and shows that caution is needed when interpreting real-world claims data due to inherent claims database limitations. As there are no specific ICD codes for VMS, difficulties exist in utilization of claims data to accurately capture VMS characteristics and treatment patterns.

## Introduction

Menopause marks the end of the reproductive years for a woman and results in varying physical manifestations. The time during which females begin transitioning into menopause is known as perimenopause.^1^ Symptoms of perimenopause typically begin in a woman’s mid to late 40s and may last for 4–8 years.^2^ Most women experience menopause between 45 and 58 years of age (average, 52 years).^2,3^ Hot flashes and night sweats are considered hallmark manifestations of menopause and are generally referred to as vasomotor symptoms (VMS).^1^ According to the Study of Women’s Health Across the Nation (SWAN), 60%–80% of US women experience VMS, and 25%–50% report 6 or more days of moderate to severe VMS symptoms within a 2-week period.^4^ Approximately half (46%) of women report the majority of their most severe hot flashes occurred during the 2 years following their final menstrual period.^5^

Race and ethnicity are the most influential factors contributing to the variation seen in the age of onset of perimenopause and prevalence and duration of VMS. During perimenopause, the prevalence of VMS is highest among African American and Hispanic women, while Chinese and Japanese women have lower rates, and White women have the lowest rates.^4^ Regardless of menopausal phase, African American women are more likely to experience VMS compared to White women (odds ratio [OR]: 1.63; *p* < 0.01).^4^ Similarly, compared to White non-Hispanic women, the adjusted VMS duration is longer for African American women (median: >10.90 years; hazard ratio [HR]: 0.75; *p* < 0.008) and Japanese women (median: 9.34 years; HR: 1.00; *p* < 0.008) and shorter for Chinese (median: 5.45 years; HR: 1.80; *p* < 0.008) and Hispanic women (median: 4.78 years; HR: 1.97; *p* < 0.008).^6^ On average, African American women enter menopause 8.5 months earlier than White women (median age: 52.17 years vs. 52.88 years, respectively; *p* = 0.0009) and have a longer duration of menopausal transition than White women.^7–9^ Similarly, Hispanic women reach menopause earlier than White women, with a median age of onset of 50.86 years (*p* = 0.0009).^9^ It is important to note that these rates are approximations and there are variations in prevalence of VMS by race from study to study, likely due to methodological issues.

Management of menopausal symptoms includes hormone therapy (HT), nonhormone treatment options (*e.g.*, selective serotonin reuptake inhibitors [SSRIs]/serotonin-norepinephrine reuptake inhibitors [SNRIs], gabapentin, oxybutynin), and nonpharmacologic interventions (*e.g.*, weight loss, clinical hypnosis, and cognitive-behavioral therapy).^10^ While HT is considered safe for short-term treatment of VMS in most people,^11^ the use of both traditionally prescribed HTs and nonhormone treatment options for VMS treatment are limited by the risk of adverse events (specifically with HT), high incidence of undesirable side effects, and questionable efficacy. With the recent Food and Drug Administration (FDA) approval of fezolinetant,^12^ a nonhormone treatment for moderate to severe VMS, this paradigm may shift, but real-world data have not matured yet for analysis.

HTs are recommended as first-line therapy in reducing VMS symptoms in women.^13^ However, despite being first-line treatments, concerns such as increased risk of venous thromboembolism, myocardial infarction, stroke, and hormone-dependent cancers (*e.g.*, breast cancer) have resulted in decreased use of HTs in VMS treatment. In the SWAN study, the initiation of HTs decreased from 8.6% to 2.8% after the 2002 Women’s Health Initiative trial was halted due to safety concerns related to HTs (increased risk of venous thromboembolism, myocardial infarction, stroke, and hormone-dependent cancers [*e.g.*, breast cancer]).^14^ Nonhormone therapies such as antidepressants (*e.g.*, SSRIs/SNRIs) and gabapentin have limited efficacy and undesirable side effects such as dizziness, nausea, and weight gain.^10^ Paroxetine, an SSRI, is the only FDA-approved SSRI for the treatment of moderate to severe VMS.^15^ In a 12-week study among patients treated with paroxetine, there was a statistically significant reduction in severity of VMS; however, this was not sustained in a 24-week study. Paroxetine use is limited by side effects including nausea, fatigue, and dizziness.^16^

Literature characterizing VMS have examined treatment patterns and symptoms; however, the research has often focused on women solely in menopause.^17,18^ Because of the variation observed in VMS manifestation and the paucity of data on VMS treatment patterns, there is a need to characterize symptomatic menopause/VMS in all menopausal phases to fully inform research gaps in this population. This retrospective claims study was conducted to evaluate the demographic and clinical characteristics of perimenopausal/menopausal and postmenopausal women with symptomatic menopause/VMS and asymptomatic menopause, the size of the population with a symptomatic menopausal state diagnosis, and the current treatment landscape of women with symptomatic menopause/VMS.

## Methods

### Data source

This retrospective cohort study utilized data from the Optum Clinformatics Datamart (CDM) Database from October 1, 2012, through September 30, 2022. The Optum CDM is composed of longitudinal individual data from a closed system of administrative health claims for members with commercial and Medicare Advantage plans that includes claims for about 67 million people from all 50 states in the United States, with data representing clinical care from a variety of real-world data sources including member, medical claims, pharmacy claims, lab test results, inpatient confinement, and provider data. Data from multiple sources are aggregated, converted to a standardized format in structured fields, and deidentified. Because of the nature of the data and deidentification, institutional review board approval was not required.

### Study objectives

The study was conducted in 2 phases. The primary objective of the study was to describe baseline demographic and clinical characteristics of perimenopausal/menopausal and postmenopausal women in the United States. The secondary objectives of the study were to estimate the size of the population with a symptomatic menopause/VMS diagnosis in the United States and to describe the current treatment landscape in perimenopausal/menopausal and postmenopausal women with symptomatic menopause/VMS in the United States.

### Patient identification

All women aged 40 − 64 years of age were considered for inclusion in both phases. Women with any diagnosis of cancer during the study period were excluded. The study period was defined as October 1, 2012, through September 30, 2022.

In Phase 1, women were classified using International Classification of Diseases (ICD)-9/10-codes as having either VMS (ICD-9: 627.7 or ICD-10: N95.1) or asymptomatic menopausal state (ICD-9: V49.81 or ICD-10: Z78.0). There is no ICD code for VMS, but as it is the most common symptom of menopause, identification of women with VMS was proxied using the ICD codes for symptomatic menopausal state.^1^

The index date was defined as the first ICD-9/10 code for symptomatic menopausal state or asymptomatic menopausal state. Age groups were chosen to proxy perimenopausal/menopausal women and postmenopausal women based on average age of onset, as there is no way to discern menopausal state in claims data. Women aged 40–54 years were classified as perimenopausal/menopausal and those aged 55–64 years were classified as postmenopausal.

In Phase 2, the study population, study period, age groups, and index date were the same as Phase 1. However, only women with newly diagnosed symptomatic menopause/VMS and continuous enrollment 6 months prior to the index date and a minimum of 12 months after the index date were included. The 6-month period prior to the index date was considered the baseline period and was used to confirm that there was no symptomatic menopause/VMS diagnosis prior to the index date. Patients with National Drug Codes for use of HT in the baseline period were excluded from Phase 2.

### Outcomes

In Phase 1, prevalence rates of symptomatic menopause/VMS and asymptomatic menopause were calculated. In this study, prevalence was defined as frequency of encounters. Baseline demographics and clinical characteristics of patients were also described for both groups. Baseline data for Phase 1 were reported yearly (2012–2022). Phase 1 values were expressed as ranges where the lower and upper values were the lowest and highest values between 2012 Q4 and 2022 Q3. Aggregated data for 2012–2022 were not available in the analysis. Prevalence rates, reported as percentages, were calculated in the overall cohort and stratified by race and ethnicity and menopausal stage (proxied by age). Prevalence rates were based on the number of asymptomatic or symptomatic menopause/VMS ICD-9/10 code instances recorded during the study period, rather than the number of unique patients. Since these total counts are aggregated across 2012–2022, ICD-9/10 codes may be counted more than once for the same patient.

In Phase 2, symptomatic menopause/VMS treatments were measured from the index symptomatic menopause/VMS diagnosis date through the earliest of end of follow-up (variable, but at minimum, 12 months), death, age 65 years, or study end date. Included treatment patterns were those noted by the North American Menopause Society guidelines for the treatment of VMS and menopausal symptoms.^10,13^ Incidence of symptomatic menopause/VMS and asymptomatic menopause, baseline demographic and clinical characteristics of patients, and treatment patterns were described. The incidence rate was calculated as the number of patients with a new symptomatic/VMS diagnosis from the year divided by the total enrollment years. The prevalence rate was calculated as the number of patients with any symptomatic/VMS diagnosis from the year divided by the total enrollment years. For asymptomatic rates, the calculations were the same, except for any patients diagnosed with both symptomatic/VMS and asymptomatic symptoms, the patient was counted in the symptomatic/VMS category. Outcomes were stratified by race and ethnicity and menopausal stage (proxied by age).

For both symptomatic menopause/VMS yearly incidence and prevalence rates, the rates were based on each of the calendar years for the women aged 40 to 64 years of age with symptomatic menopause/VMS. For each calendar year, the total enrollment year was the sum of all enrolled days from the patient population (insured divided by 365). If a patient was enrolled for the full year, the patient was counted as having 1 total enrollment year. If a patient was enrolled for 3 months, that patient was counted as having only a quarter total enrollment year.

### Statistical analysis

Descriptive statistics (*e.g.*, percentages, means, medians, standard deviations) were used to describe all study outcomes. No statistical tests were performed. Women were censored at the age of 65 years with no additional follow-up. Prevalence and incidence of symptomatic menopause/VMS were calculated as a percentage in the overall cohort and stratified by race and ethnicity and menopausal stage (perimenopausal/menopausal vs. postmenopausal).

## Results

### Phase 1

#### Baseline characteristics

A total of 9,877,282 women aged 40–64 years were identified during the study period in which a total of 1,987,355 ICD-9/10 codes for symptomatic menopause/VMS or asymptomatic menopause were used for prevalence calculations. From 2012 to 2022, there were similar proportions of identified women with ICD-9/10 codes for asymptomatic or symptomatic menopause/VMS in the perimenopausal/menopausal and postmenopausal states per year (ranges: 46.2%−54.1% and 45.9%−53.8%, respectively). The mean (standard deviation [SD]) age by year ranged from 53.8 (5.7) years to 54.9 (5.7) years. Most women were White, with the proportion ranging from 70.4% to 76.9% per year over the study period. The mean (SD) Charlson Comorbidity Index (CCI) score ranged from 0.4 (1.0) to 0.6 (1.4) per year (see Table 1 for more details on baseline demographics).

**Table 1.** Patient Demographics

Characteristics	Phase 1^a^		
	Patient groups		
	All patients (*N* = 1,987,355)^b^	Symptomatic (*n* = 1,350,873)^b^	Asymptomatic (*n* = 636,482)^b^
Age (mean, SD)	53.8 (5.7); 54.9 (5.7)	53.0 (5.6); 53.4 (5.6)	56.9 (4.9); 57.7 (5.0)
Age group (*n*, %)			
Age 40–54	32,137 (54.1); 99,652 (49.0)	28,411 (60.4); 90,740 (59.9)	3,726 (30.3); 20,483 (27.2)
Age 55–64	27,254 (45.9); 114,980 (53.8)	18,663 (39.6); 60,816 (40.1)	8,591 (69.7); 57,976 (74.1)
Race (*n*, %)			
Asian	1,487 (2.5); 6,333 (3.1)	1,123 (2.4); 3,766 (2.5)	364 (3.0); 2,745 (3.6)
Black	5,163 (8.7); 19,296 (9.0)	4,162 (8.8); 14,385 (9.5)	1,001 (8.1); 7,227 (9.2)
Hispanic	4,568 (7.7); 22,218 (10.4)	3,777 (8.0); 14,482 (10.7)	791 (6.4); 7,736 (9.9)
White	45,648 (76.9); 155,069 (72.5)	36,024 (76.5); 114,928 (75.8)	9,624 (78.1); 56,629 (72.4)
Region (*n*, %)			
Midwest	13,561 (22.8); 46,087 (21.6)	9,432 (20.0); 30,815 (20.3)	4,129 (33.5); 21,029 (26.9)
Northeast	3,777 (6.4); 16,326 (7.6)	2,754 (5.9); 9,150 (6.0)	1,023 (8.3); 8,134 (10.4)
South	28,631 (48.2); 105,914 (49.5)	23,965 (50.9); 76,304 (50.3)	4,666 (37.9); 34,671 (46.1)
West	13,387 (22.5); 46,259 (21.9)	10,895 (23.1); 35,157 (23.2)	2,492 (20.2); 14,861 (19.6)
CCI (mean, SD)	0.4 (1.0); 0.6 (1.4)	0.4 (0.9); 0.5 (1.1)	0.6 (1.2); 0.9 (1.7)

Characteristics	Phase 2				
	Patient groups				
	All patients (*N* = 382,204)	Symptomatic (*n* = 263,815)	Asymptomatic (*n* = 118,389)	Treated (*n* = 202,101)	Un-treated (*n* = 180,103)
Age (mean, SD)	53.9 (5.9)	52.4 (5.6)	57.3 (4.9)	53.8 (5.8)	54.1 (5.9)
Age group (*n*, %)					
Age 40–54	203,546 (53.3)	172,097 (65.2)	31,449 (26.6)	109,931 (54.4)	93,615 (52.0)
Age 55–64	178,658 (46.7)	91,718 (34.8)	86,940 (73.4)	92,170 (45.6)	86,488 (48.0)
Race (*n*, %)					
Asian	13,297 (3.5)	8,513 (3.2)	4,784 (4.0)	4,418 (2.2)	8,879 (4.9)
Black	33,375 (8.7)	23,132 (8.8)	10,243 (8.7)	15,735 (7.8)	17,640 (9.8)
Hispanic	40,063 (10.5)	28,941 (11.0)	11,122 (9.4)	18,700 (9.3)	21,363 (11.9)
White	282,654 (74.0)	194,505 (73.7)	88,149 (74.5)	156,790 (77.6)	125,864 (69.9)
Region (*n*, %)					
Midwest	97,700 (25.6)	59,683 (22.6)	38,017 (32.1)	49,044 (24.3)	48,656 (27.0)
Northeast	30,856 (8.1)	17,953 (6.8)	12,903 (10.9)	14,427 (7.1)	16,429 (9.1)
South	170,094 (44.5)	125,553 (47.6)	44,541 (37.6)	94,488 (46.8)	75,606 (42.0)
West	82,977 (21.7)	60,190 (22.8)	22,787 (19.2)	43,938 (21.7)	39,039 (21.7)
CCI (mean, SD)	1.4 (2.1)	1.2 (2.0)	1.6 (2.4)	1.5 (2.3)	1.1 (1.9)
Comorbidities (*n*, %)					
Chronic pulmonary disease	95,782 (25.1)	64,355 (24.4)	31,427 (26.5)	59,322 (29.4)	36,460 (20.2)
Depression	122,190 (32.0)	87,425 (33.1)	34,765 (29.4)	93,105 (46.1)	29,085 (16.1)
Hypertension	173,317 (45.3)	112,002 (42.5)	61,315 (51.8)	97,502 (48.2)	75,815 (42.1)
Insomnia	92,022 (24.1)	69,189 (26.2)	22,833 (19.3)	67,326 (33.3)	24,696 (13.7)
Joint pain	221,427 (57.9)	153,513 (58.2)	67,914 (57.4)	128,895 (63.8)	92,532 (51.4)
Mood disorders	128,315 (33.6)	91,804 (34.8)	36,511 (30.8)	97,169 (48.1)	31,146 (17.3)
Obesity	108,207 (28.3)	73,457 (27.8)	34,750 (29.4)	60,178 (29.8)	48,029 (26.7)
Other sleep disturbances	1,104 (0.3)	778 (0.3)	326 (0.3)	818 (0.4)	286 (0.2)

^a^ Phase 1 values are expressed as ranges where the lower and upper values are the lowest and highest values between 2012 Q4 and 2022 Q3.

^b^ Total count (*i.e.*, N or n) is not the number of unique patients. Rather, it is representative of how many asymptomatic or symptomatic menopause ICD-9/10 code instances were recorded during the study period. Since these total counts are aggregated across 2012–2022, ICD-9/10 codes may be counted more than once for the same patient.

CCI, Charlson comorbidity index; ICD, International Classification of Diseases; NA, not available; SD, standard deviation.

#### Prevalence

Among women identified in the menopausal state, symptomatic menopause/VMS prevalence was 5.72% (95% confidence interval [CI]: 5.71–5.73), and asymptomatic perimenopause/menopause prevalence was 2.78% (95% CI: 2.77–2.79). Symptomatic menopause/VMS prevalence by year from 2012 to 2022 ranged from 4.93% to 7.99% and asymptomatic perimenopause/menopause prevalence ranged from 2.04% to 3.40%.

White women had a higher symptomatic menopause/VMS prevalence (6.26%; 95% CI: 6.23–6.27) compared to Hispanic (4.98%; 95% CI: 4.96–5.01), Black (4.59%; 95% CI: 4.57–4.62), and Asian (3.38; 95% CI: 3.34–3.41) women. In the perimenopausal/menopausal group, White women had the highest symptomatic menopause/VMS prevalence (7.27%; 95% CI: 7.25–7.28) while Asian women had the lowest (3.52%; 95% CI: 3.48–3.57). When looking at symptomatic menopause/VMS prevalence data by age group, perimenopausal/menopausal women had a higher symptomatic menopause/VMS prevalence compared to postmenopausal women (6.50% vs. 4.87%).

Similar to symptomatic menopause/VMS prevalence, White women had a higher prevalence of asymptomatic menopause (3.03%; 95% CI: 3.02–3.03) compared to Black (2.29%; 95% CI: 2.27–2.31), Hispanic (2.22%; 95% CI: 2.21–2.24), and Asian women (2.10%; 95% CI: 2.07–2.12). Across all race and ethnicity groups, the asymptomatic menopausal prevalence was higher in postmenopausal women compared to perimenopausal/menopausal women (4.15% [95% CI: 4.14–4.16] vs. 1.45% [95% CI:1.4–1.46]). In postmenopausal women, White women had the highest asymptomatic prevalence (4.36%; 95% CI: 4.35–4.38), while Black women had the lowest asymptomatic prevalence (3.41%; 95% CI: 3.38–3.44).

### Phase 2

#### Baseline characteristics

Of the 9,877,282 women identified during the study period aged 40–64 years, 382,204 women were identified with newly diagnosed menopause and met the inclusion criteria. Rates of perimenopausal (53.3%; *n* = 203,546) versus postmenopausal (46.7%; *n* = 178,658) status were similar. A total of 69% (*n* = 263,815) of women from the total study population were classified as symptomatic, while 31.0% (*n* = 118,389) were asymptomatic. In addition, 52.9% (*n* = 202,101) of women were treated while 47.1% (*n* = 180,103) were untreated. The mean (SD) age was 53.9 (5.9) years. Most patients were White (74.0%; *n* = 282,654) and the mean (SD) CCI was 1.4 (2.1). Comorbidities of interest based on their potential association with menopause included joint pain (57.9%; *n* = 221,427), mood disorders (33.6%; *n* = 128,315), depression (32.0%; *n* = 122,190), insomnia (24.1%; *n* = 92,022), and other sleep disturbances (0.3%; *n* = 1,104). Other comorbidities reported in >20% of the total study population included hypertension (45.3%; *n* = 173,317), obesity (28.3%; *n* = 108,207), and chronic pulmonary disease (25.1%; *n* = 95,782) (see Table 1 for more details on baseline demographics).

#### Incidence

The overall incidence of menopause from 2012 to 2022 was 1.69% (95% CI: 1.68, 1.69), including 1.17% (95% CI: 1.17, 1.18) with symptomatic menopause/VMS and 0.53% (95% CI: 0.53, 0.53) with asymptomatic menopause. The incidence of symptomatic menopause/VMS when comparing across race and ethnicity was highest in White women (1.29%; 95% CI: 1.28–1.29), followed by Hispanic (1.10%; 95% CI: 1.09–1.11), Black (0.92%; 95% CI: 0.91–0.93), and Asian (0.82%; 95% CI: 0.81–0.84) women.

The incidence of asymptomatic menopause was highest in White women (0.59%; 95% CI: 0.58–0.59), followed by Asian (0.46%; 95% CI: 0.45–0.49), Hispanic (0.43%; 95% CI: 0.42–0.43), and Black women (0.41%; 95% CI: 0.40–0.42). Symptomatic menopause/VMS incidence was higher for perimenopausal/;menopausal women compared to postmenopausal women (1.48% [95% CI: 1.47–1.49] vs. 0.84% [95% CI: 0.83–0.85]); however, when comparing incidence of asymptomatic menopause, the opposite trend was observed, where postmenopausal women had a higher incidence of asymptomatic menopause compared with perimenopausal/menopausal women (0.80% [95% CI: 0.79–0.80] vs. 0.27% [95% CI: 0.27–0.28]).

#### Treatment patterns

Among the total population, 52.9% (*n* = 202,101/382,204) were treated for symptomatic menopause/VMS compared to 47.1% (*n* = 180,103/382,204) who were untreated (*i.e.*, those who were not using any pharmacologic therapies to manage symptomatic menopause/VMS). Within the treated group, perimenopausal/menopausal women had a higher proportion treated compared to postmenopausal women (54.4% [*n* = 109,931/202,101] vs. 51.6% [*n* = 92,170/202,101], respectively). The percentage of women treated for symptomatic menopause/VMS within each race and ethnicity was as follows: White (55.5%; *n* = 156,790/282,654), Hispanic (46.7%; *n* = 18,700/40,063), Black (47.1%; *n* = 15,735/33,375), and Asian (33.2%; *n* = 4,418/13,297).

In perimenopausal/menopausal women (*N* = 109,931), the count of post-index outpatient visits was higher in treated than untreated women (109,643 vs. 92,633, respectively). When considering 3 drug classes (HT, SSRI/SNRI, and insomnia medications), for treated women, the mean (SD) number of drug classes used was 1.5 (0.6) and the median (range) days to discontinuation was 222 (1, 3,293) days, with 38.2% (*n* = 41,972/109,931) of patients using 2 or more drug classes for symptomatic menopause/VMS. Among perimenopausal/menopausal treated women, 37.3% (*n* = 40,977/109,931) were using HT, 29.7% (*n* = 32,618/109,931) were using SSRIs/SNRIs, and 33.0% (*n* = 36,311/109,931) were using insomnia medications. Perimenopausal/menopausal women treated with insomnia medications had a lower mean (SD) compliance (0.6 [0.4]) compared with those treated with HT (0.8 [0.3]) and SSRIs/SNRIs (0.8 [0.2]). The 1-year medication possession ratio was also lower among patients treated with insomnia medication, and days to discontinuation for insomnia medication were reached sooner compared to those treated with SSRIs/SNRIs. The discontinuation rates across all 3 drug classes were greater than 50% (HT: 67.6%; SSRI/SNRI: 57.2%; insomnia medications: 77.8%) (see Table 2 for more details on discontinuation rates).

**Table 2.** Treatment Trends by Menopausal State and Treatment Type for Treated Patients (N = 202,101)

Menopausal state	Treatment, *n* (%)	Number of drug classes used, *n* (%)			Compliance^a^, mean (SD)	1-year MPR, mean (SD)	Count of post-index outpatient visits, median (range)	Total follow-up days, median (range)	Index drug DC’d, *n* (%)	Days to discontinuation, median (range)
		1	2	3						
Peri/ menopausal (age 40–54) *N* = 109,931	HT, 40,977 (37.3)	26,332 (64.3)	10,890 (26.6)	3,755 (9.2)	0.8 (0.3)	0.6 (0.4)	26 (1.0–1,029.0)	853 (91.0–3,469)	27,694 (67.6)	210 (5.0–3,219.0)
	SSRI/SNRI, 32,618 (29.7)	19,164 (58.8)	11,087 (34.0)	2,367 (7.3)	0.8 (0.2)	0.7 (0.4)	26 (1.0–1,219.0)	804 (91.0–3,469.0)	18,670 (57.2)	254 (7.0–3,192.0)
	Insomnia medication, 36,311 (33.0)	22,450 (61.8)	11,497 (31.7)	2,364 (6.5)	0.6 (0.4)	0.4 (0.4)	34 (1.0–1,098.0)	870 (92.0–3,469.0)	28,237 (77.8)	215 (1.0–3,293.0)
Postmenopausal (age 55–64) *N* = 92,170	HT, 37,102 (40.3)	24,393 (65.7)	9,802 (26.4)	2,907 (7.8)	0.8 (0.3)	0.6 (0.4)	27 (1.0–1,320.0)	805 (92.0–3,469.0)	25,060 (67.5)	204 (1.0–3,176.0)
	SSRI/SNRI, 24,392 (26.5)	14,599 (59.9)	8,138 (33.4)	1,655 (6.8)	0.9 (0.2)	0.8 (0.3)	28 (1.0–882.0)	764 (92.0–3,466.0)	11,689 (47.9)	270 (10.0–3,292.0)
	Insomnia medication, 30,660 (33.3)	19,617 (64.0)	9,391 (30.6)	1,652 (5.4)	0.7 (0.4)	0.5 (0.4)	36 (1.0–1,239.0)	824 (91.0–3,469.0)	22,473 (73.3)	214 (1.0–3,331.0)

^a^ Compliance was calculated as the sum of all supply days (not counting the last fill) and dividing it by the difference between last fill date and index date. If a patient had only 1 fill, the compliance value was set to null.

DC’d, discontinued; HT, hormone therapy; MPR, medication possession ratio; SNRI, serotonin and norepinephrine reuptake inhibitor; SSRI, selective serotonin reuptake inhibitor.

In postmenopausal women (*N* = 92,170), the count of post-index outpatient visits was higher in treated than untreated patients (91,988 vs. 85,793, respectively). For the treated group, the mean (SD) number of drug classes used was 1.4 (0.6) and the median (range) days to discontinuation was 221 (1.0, 3,331) days, with 36.4% (*n* = 33,551/92,170) of patients using two or more drug classes for symptomatic menopause/VMS. Among postmenopausal women, 40.3% (*n* = 37,102/92,170) were using HT, 26.5% (*n* = 24,392/92,170) were using SSRIs/SNRIs, and 33.3% (*n* = 30,660/92,170) were using insomnia medications. There was a slightly higher utilization of HT among postmenopausal women compared to perimenopausal/menopausal women (40.3% vs. 37.3%). Postmenopausal women treated with insomnia medication experienced a similar trend as perimenopausal/menopausal women treated with insomnia medications (Table 2). The discontinuation rates across all three drug classes in postmenopausal women were greater than 48% (HT: 67.5%; SSRI/SNRI: 47.9%; insomnia medications: 73.3%).

## Discussion

In this study, both prevalence and incidence of symptomatic menopause/VMS were observed to be higher in perimenopausal/menopausal women aged 40–54 years compared to postmenopausal women aged 55–64 years across all race and ethnicity groups. While this trend with advancing menopausal status coincides with previously reported patterns, prevalence rates observed in this study differed from the published literature in several key aspects. Overall US prevalence of symptomatic menopause/VMS has been reported as ranging anywhere from 25% to 80%.^4^ In marked contrast, the symptomatic menopause/VMS prevalence in the data analyzed for this study was only 5.7%. When looking at symptomatic menopause/VMS prevalence by race and ethnicity, which are socioeconomic constructs utilized to examine racial differences in this study, higher symptomatic menopause/VMS rates have been previously reported in African American and Hispanic women, with lower rates seen among Chinese and Japanese women compared to White women.^4^ This trend of symptomatic menopause/VMS rates by race and ethnicity was not reflected in our study, where White women were reported to have the highest prevalence of symptomatic menopause/VMS at 8.9%, followed by Hispanic and Black women (7.0% and 6.7%, respectively), with Asian women having the lowest reported VMS rates at 5.3%.

Reasons as to why prevalence rates in this study were lower compared to what has been reported in existing literature could be most likely due to the fact that many women who experience VMS do not pursue medical treatment specifically for those symptoms, as previously documented in multiple surveys among menopausal women experiencing VMS.^19–24^ Additional reasons include underreporting among racial and ethnic minority groups, health care provider unfamiliarity of symptomatic menopause/VMS manifestations among various racial and ethnic minority groups, lack of coding due to patients presenting for other reasons for care, treatment access barriers, or the patient population included in the Optum CDM. Underreporting among racial and ethnic minority groups may be prevalent, as these groups are less likely to have symptoms documented and receive treatment.^25^ Women who need to be treated for symptomatic menopause/VMS may not have access to health care, especially among racial and ethnic minority groups, and therefore symptomatic menopause/VMS would not be documented in claims data.^26^ Health care providers may be unfamiliar with the differences in symptomatic menopause/VMS manifestations at different time points depending on race and ethnicity and therefore might not know when certain symptoms are related to symptomatic menopause/VMS versus another medical condition.^25^ Lastly, Optum CDM included health care claims from commercial and Medicare Advantage plans. Women on Medicaid and those who are uninsured were not included as part of this study.

Treatment trends were captured in Phase 2 of this study. In this study, about 47% (*N* = 382,204) of patients were untreated after symptomatic menopause/VMS diagnosis, which has been observed to be comparable or even higher in the published literature.^11,17^ Patients are reluctant to seek treatment for symptomatic menopause/VMS due to a variety of reasons, including general desire to avoid prescription therapies, concerns with long-term safety of HT due to increased risk of known complications, including cardiovascular complications (*e.g.*, stroke, myocardial infraction, venous thromboembolism), and efficacy and tolerability of SSRIs/SNRIs.^14,17^

Among treated patients in this study, HT utilization was about 40%; however, discontinuation rates were high at approximately 70% across perimenopausal/menopausal and postmenopausal age groups. Despite being an effective treatment option, many symptomatic women do not choose to use HT for a variety of reasons, including perceived safety concerns.^17^ Insomnia medications had the lowest compliance and highest discontinuation rates among perimenopausal/menopausal and postmenopausal women compared to HT and SSRI/SNRI medications. Rates of discontinuation across all 3 drug classes studied (HT, SSRIs/SNRIs, and insomnia medications) were approximately 50% or greater. This observed high treatment discontinuation rate could be the result of undesirable side effects or limited efficacy of current symptomatic menopause/VMS treatments. Limited efficacy of current treatments may be reflected in this study as 33% of patients were using more than 1 medication class to potentially treat their symptomatic menopause/VMS-related symptoms. Novel treatments could offer alternative choices for women experiencing bothersome symptomatic menopause/VMS by exhibiting improved efficacy and a more favorable side effect profile.^10,14^

Patients frequently report the negative impact of symptomatic menopause/VMS on their quality of life.^21^ Patients who experience frequent symptomatic menopause/VMS (*i.e.*, >6 days in 2 weeks) have reported higher rates of comorbidities and have an overall reported decreased quality of life.^11^ As seen in this study, >20% of patients had comorbidities such as chronic pulmonary disease, depression, hypertension, and insomnia, which can lead to undesirable long-term health outcomes. Patients with symptomatic menopause/VMS often delay seeking care and remain untreated, despite reporting bothersome symptoms.^18^ Untreated symptomatic menopause/VMS are associated with higher health care resource use, such as outpatient visits, and higher treatment-associated costs.^27^

Other pharmacological treatments for symptomatic menopause/VMS include SSRIs/SNRIs and gabapentin. While the SSRI paroxetine has been approved by the FDA for VMS treatment,^15^ the results of placebo-controlled clinical trials for SSRIs/SNRIs or gabapentin have demonstrated inconsistent or short-term effectiveness in decreasing symptomatic menopause/VMS frequency or severity.^16,28,29^ Studies with gabapentin or SSRIs/SNRIs have also reported increased adverse events leading to higher discontinuation rates relative to placebo.^16,28,30^ Due to high discontinuation rates and compounded effects of comorbidities, there is an unmet need for new therapies that have minimal side effects while being effective in treating symptomatic menopause/VMS.

### Limitations

The use of a claims database for analysis introduces inherent limitations. Although the Optum CDM Database includes beneficiaries from a large national health plan, members may not be fully representative of the affected US population. In addition, only women with moderate to severe symptomatic menopause/VMS are likely to seek care and receive a documented diagnosis, which may result in underestimates of prevalence and incidence.

Another limitation associated with this claims database study include lack of ICD-9/10 codes specific to VMS. Therefore, symptomatic menopause claims were used as a proxy for the presence of VMS, as symptoms such as hot flashes are reported to be the hallmark of VMS.^1^ However, it is possible that some women included in the symptomatic group were only experiencing non-VMS symptoms. In addition, the indication for drug prescriptions was unavailable in the dataset so it could not be confirmed which treatments were used specifically to treat VMS symptoms.

Regarding limitations related to the methods, this study did not identify or report any social determinants of health, which could have added another layer of granularity and context to the racial differences reported in the results. Further, we assumed that symptomatic menopause was equivalent to VMS due to the lack of an ICD code, which may have led to a low prevalence of VMS inconsistent with the literature. Other literature identified women with VMS through self-reported presence and frequency of hot flashes or night sweats in the prior 2 weeks,^4^ which would have included a greater number of patients than the ICD codes for symptomatic menopause. However, since this study utilized a claims database, the use of alternative ICD codes to identify VMS were required for data analysis.

Patients may also have been misclassified as perimenopausal/menopausal or postmenopausal in this study, as the classification of menopausal status as a diagnosis has not been systematically tested with standardized documentation in patient health care profiles. Based on prior longitudinal studies, age ranges representing menopausal status were chosen to define perimenopausal/menopausal (ages 40–54 years) and postmenopausal (ages 55–64 years) groups.^9^

## Conclusion

This study adds important information to the literature regarding the characteristics of women with symptomatic menopause/VMS across the menopausal spectrum by ethnicity and age, such as the prevalence and incidence of symptomatic menopause/VMS diagnoses, treatment patterns, and rates of selected comorbidities relevant to symptomatic menopause/VMS. However, it also illustrates that the use of claims data may not always reflect the true breadth and depth of population characteristics. For example, as seen with this study, lower rates of frequent and severe menopausal symptoms were observed in both Black and Hispanic women, which is contrary to what has been reported in the published literature. This difference may be due to underreporting from both physicians and patients among racial and ethnic minority groups and other factors such as misclassification due to lack of VMS-specific ICD-9/10 codes. Additionally, as there are no specific ICD codes for VMS, difficulties exist in utilization of claims data to accurately capture VMS characteristics and treatment patterns. It is key to consider these factors and use caution when interpreting real-world claims data results. While the results from this study have provided more descriptive insights into women with symptomatic menopause/VMS, they have also highlighted potential disparities of care, underreporting of symptomatic menopause/VMS symptoms.

## Abbreviations Used

CCI: Charlson Comorbidity Index
CDM: Clinformatics Datamart
CI: confidence interval
FDA: Food and Drug Administration
HR: hazard ratio
HT: hormone therapy
ICD 9/10: International Classification of Diseases, Ninth/Tenth Revision
NDC: National Drug Code
OR: odds ratio
SD: standard deviation
SNRI: serotonin-norepinephrine reuptake inhibitor
SSRI: selective serotonin reuptake inhibitor
SWAN: Study of Women’s Health Across the Nation
US: United States
VMS: vasomotor symptoms
